# Socio-Ecological Correlates of Food Literacy Across Regional Contexts in China

**DOI:** 10.3390/nu18071151

**Published:** 2026-04-03

**Authors:** Yingying Li, Ji-Yun Hwang

**Affiliations:** 1 Department of Foodservice Management and Nutrition, Graduate School, Sangmyung University, Seoul 03016, Republic of Korea; liy407853@gmail.com; 2 Major of Foodservice Management and Nutrition, Sangmyung University, Seoul 03016, Republic of Korea

**Keywords:** food literacy, socio-ecological determinants, regional heterogeneity, China

## Abstract

Background: Food literacy (FL) comprises the knowledge, skills, and motivation needed for food production, selection, preparation, intake, and waste management. This study examined whether socio-ecological correlates of FL differ across settlement contexts in China. Methods: Cross-sectional survey data from Chinese adults (*N* = 1145) were analyzed across four settlement tiers: tier-1 metropolitan cities (R1), provincial/secondary cities (R2), smaller prefecture-level cities (R3), and county/rural areas (R4). General linear models estimated associations between socio-ecological predictors and overall FL after adjustment for sociodemographics, health behaviors, chronic disease, and BMI. Significant interactions were probed using HC3-robust simple slopes and pairwise slope contrasts. Robustness checks included domain-specific measurement invariance, variance inflation factor (VIF) diagnostics, and a regional sensitivity analysis. Results: The fully adjusted model explained substantial variance in FL (R^2^ = 0.629). Awareness showed the strongest association with FL, followed by family support, injunctive norms, and social norms. Moderation was modest and predictor-specific: dining preferences and family support were positively associated with FL across all regions, with the strongest effects in county/rural areas. Although the omnibus interaction for injunctive norms was statistically significant, follow-up slope contrasts were not, indicating limited substantive regional heterogeneity. Component analyses indicated that preference-related heterogeneity was concentrated in food intake and food choices/selection, whereas family-support heterogeneity was most pronounced for waste disposal. Domain-level invariance analyses supported broad cross-regional comparability of the FL structure, VIFs were all below 5, and the regional distribution of valid and invalid responses did not differ significantly. Conclusions: Socio-ecological correlates of FL were broadly robust across China, with limited context-specific variation driven mainly by stronger household-support effects in county/rural settings. These findings support region-sensitive FL strategies that strengthen household-based support while leveraging normative influences across regions.

## 1. Introduction

China faces a rapidly evolving nutritional landscape marked by rising diet-related chronic disease and persistent gaps between recommended and actual dietary patterns [1,2,3,4]. Rapid urbanization, market transition, and changing food environments have expanded access to processed foods, delivery platforms, and modern retail, while also reshaping affordability, convenience, and social eating practices [1,2,3,4].

Food literacy (FL) has emerged as a promising upstream determinant of healthier eating because it captures the knowledge, skills, and confidence needed to plan, select, prepare, eat, and manage food in daily life [5,6,7,8,9,10]. Higher FL has been linked to better diet quality in diverse adult populations, including Korea, Japan, and Europe, suggesting both public health relevance and cross-context validity [11,12,13].

Socio-ecological theory suggests that FL is shaped not only by intrapersonal factors, but also by interpersonal support and broader food-related environments [14,15,16,17]. In China, these influences may not operate uniformly across settlement contexts. Differences in retail access, digital food environments, family structure, mobility, and local food culture across metropolitan, secondary-city, smaller-city, and county/rural settings may alter how preferences, family support, and norms translate into FL [3,14,15,16,17].

Despite growing interest in FL, relatively few studies have modeled socio-ecological determinants of FL within a single analytic framework, and even fewer have tested whether these relationships vary across China’s diverse regional contexts [5,6,7,8,9,10,11,12,13]. This leaves an important gap in evidence needed to design region-sensitive interventions.

This study, therefore, examined socio-ecological correlates of FL among Chinese adults and tested whether their associations differed across four regional tiers. Guided by socio-ecological theory, we considered intrapersonal, interpersonal, and environmental determinants of FL and explicitly tested regional moderation. In this study, “rural amplification” refers to a pattern in which the association between a socio-ecological determinant and FL is stronger in county/rural settings than in metropolitan settings, consistent with evidence that support-related processes can differ between rural and urban China [18]. Recent work in Chinese adult samples has also linked FL to healthy eating intentions, underscoring the value of extending this research toward upstream socio-ecological determinants [19,20].

## 2. Materials and Methods

### 2.1. Study Design and Ethical Approval

This study is a secondary analysis of a cross-sectional survey of Chinese adults based on the same survey dataset used in prior work [20]. This cross-sectional survey covers the adult population in four major categories of residential areas in Henan and Shandong provinces: first-tier cities (metropolitan cities), provincial capitals and second-tier cities, medium and small cities, as well as county and rural regions. Data collection occurred from October 2023 to March 2024. The study received institutional review board approval from Sangmyung University (IRB approval numbers: SMUIRBC-2023-001 [23 June 2023] and SMUIRBC-2024-024 [12 June 2024]).

### 2.2. Participants and Sampling

Eligible participants were adults aged 18–64 years who were residing in mainland China at the time of the survey. Using a snowball sampling approach, questionnaires were distributed via QR codes and WeChat links. A total of 1249 Chinese adults completed the survey, which included an incentive. To ensure data quality, we applied five pre-specified screening criteria tailored to the length and structure of the questionnaire, which included multiple matrix-based scales and repeated behavioral items across modules. Responses were excluded if they (1) were incomplete or had excessive missingness on key constructs (e.g., FL and TPB-related scales), (2) had implausibly short completion times indicative of speeding, (3) exhibited non-differentiated response patterns (e.g., straight-lining/low within-scale variance) in matrix blocks, (4) showed substantial inconsistencies across conceptually overlapping items measured in different sections (e.g., fruit/vegetable intake and nutrition label use), or (5) contained implausible values or logical contradictions in demographic/health information (e.g., sex-inconsistent pregnancy/lactation responses). Applying these criteria, we excluded 104 respondents, yielding a final analytic sample of 1145 eligible participants. Sampling quotas ensured adequate coverage across the four regional categories.

### 2.3. Conceptual Framework

Guided by socio-ecological theory, this study conceptualizes FL as the outcome of layered influences across intrapersonal, interpersonal, and environmental domains, and explicitly tests whether these associations vary by region within China [21,22]. We focus on: (1) intrapersonal factors such as dining preferences and FL-related awareness; (2) interpersonal factors, including family support and injunctive norms; and (3) environmental factors, such as perceived social norms and family food availability. We further treat region (tier-1 cities, provincial/secondary cities, smaller cities, county/rural areas) as a contextual moderator that may strengthen or attenuate the impact of these determinants on FL.

Accordingly, our conceptual framework posits that:

**Hypothesis** **1 (H1).** *Higher levels of supportive intrapersonal, interpersonal, and environmental factors are associated with higher FL.*

**Hypothesis** **2 (H2).** *These associations vary by regional context, reflecting differential opportunities, constraints, and normative climates across socio-spatial settings. In this study, “rural amplification” refers to a pattern in which the association between a socio-ecological determinant and FL is stronger in county/rural settings than in metropolitan settings, consistent with prior evidence that support-related processes can differ between rural and urban contexts in China [18].*

### 2.4. Survey Instrument

#### 2.4.1. FL

FL was assessed using a multidimensional instrument originally developed in Korea and conceptually grounded in the domains proposed by Vidgen and Gallegos and subsequent literature [5,7]. The scale covers five components reflecting key competencies across the food system: (1) production (e.g., basic food sourcing and safety), (2) selection/choices (e.g., reading and using food information), (3) preparation/cooking, (4) intake (e.g., balanced eating in daily life), and (5) disposal/waste management. The instrument consists of 25 items rated on a five-point Likert scale, with higher scores indicating higher self-perceived FL [23].

For use among mainland Chinese adults, all items were translated and culturally adapted through forward translation, expert panel review in nutrition and public health, and a small pilot test to ensure clarity and contextual relevance while retaining the original domain structure and response options. The same adapted version and dataset were previously used to examine associations between FL and healthy eating intentions among Chinese adults [20]. In that work, domain-specific internal consistency was acceptable to excellent (Cronbach’s α approximately 0.80–0.90 across the five components), supporting the reliability of the instrument in this population. Given that the current study is based on the same survey dataset, those reliability estimates apply directly; the present analysis therefore focuses on the socio-ecological correlates and regional moderation of FL rather than on revalidating the scale.

FL was scored following the procedure recommended by the original scale developers [23]. Each item was rated from 0 (not at all) to 4 (very much so). First, item scores were multiplied by their designated item weights. Second, for each of the five domains—production, selection/choices, preparation/cooking, intake, and disposal—the weighted item scores were summed to obtain a domain score. Third, domain scores were multiplied by the corresponding domain weights and summed to generate an overall FL index. Higher domain and total scores indicate greater food-related knowledge, skills, confidence, and responsible practices across the continuum from food planning and procurement to consumption and waste management [23].

#### 2.4.2. Socio-Ecological Determinants

Socio-ecological determinants were organized at three levels—intrapersonal, interpersonal, and environmental—following ecological models of health behavior and food environments [14,15,16,17]. All items were rated on five-point Likert scales, with higher scores indicating stronger endorsement. Item pools for motivation-related constructs were informed by prior work in Chinese adults and were refined for the present study through expert review and pilot testing [20,24].

At the intrapersonal level, the study assessed dining preferences, perceived importance of healthy eating, agreement with healthy-eating beliefs, and food-related awareness. At the interpersonal level, injunctive norms and family support were measured. At the environmental level, perceived social norms and household healthy food availability were assessed. 

Reliability and convergent validity were acceptable across constructs (Cronbach’s α = 0.824–0.917, CR ≥ 0.828, and AVE = 0.546–0.706), supporting the use of these variables in the subsequent moderation analyses.

Across all socio-ecological constructs, the measurement results supported their treatment as continuous predictors in the multivariable models.

### 2.5. Other Variables

Region was coded into four categories—tier-1 metropolitan cities (R1), provincial/secondary cities (R2), smaller prefecture-level cities (R3), and county/rural areas (R4)—based on administrative level, economic development, and population size. This tiered classification is widely adopted in the Chinese population and market research to capture structural differences in economic resources, urban infrastructure, and food environments, and aligns with documented regional disparities in diet, nutrition transition, and chronic disease burden in China [1,2,3,4]. In this study, the region serves as the focal contextual moderator to test whether socio-ecological determinants of FL operate differently across heterogeneous living environments.

A priori covariates included age, sex, marital status, education, occupation, household income, BMI, chronic disease, smoking, alcohol use, and physical activity. These variables were included as potential confounders of the associations between socio-ecological determinants, region, and FL [2,3,4], and were treated as adjustment variables rather than as primary explanatory factors.

### 2.6. Instrument Adaptation, Reliability, and Validity

The socio-ecological measurement model demonstrated acceptable-to-good fit (χ^2^(436) = 1615.37, χ^2^/*df* = 3.71; RMSEA = 0.049, 90% CI = 0.046–0.051; SRMR = 0.050; CFI = 0.947; TLI = 0.940; NFI = 0.930; IFI = 0.948; GFI = 0.916). Although AGFI (0.899) marginally missed the conventional cutoff and RMR (0.098) was relatively high, these were accompanied by satisfactory incremental and residual-based indices, supporting overall model adequacy according to commonly used methodological guidance [25,26,27].

### 2.7. Statistical Analysis

We first summarized continuous variables as mean ± SD and categorical variables as *n* (%), and assessed scale reliability using Cronbach’s α, composite reliability (CR), and average variance extracted (AVE).

Regional differences in baseline characteristics were examined using chi-square tests for categorical variables and general linear models or one-way ANOVA for continuous variables, with Cramér’s V used to summarize effect size for categorical comparisons.

We then fitted general linear models to examine associations of socio-ecological determinants with overall FL and the five FL components, adjusting for the prespecified covariates. The region was modeled as a four-category factor (R1–R4).

Regional moderation was tested by adding Region × predictor interaction terms. When an interaction was significant, conditional effects were probed using PROCESS Model 1 with HC3 robust standard errors to estimate simple slopes and pairwise slope contrasts.

Additional robustness checks included domain-specific measurement invariance analyses across R1–R4, interpreted using changes in CFI and RMSEA across nested models [28], variance inflation factor (VIF) diagnostics for the fully adjusted model [29], and a sensitivity analysis comparing the regional distribution of valid and invalid responses. All analyses were conducted in IBM SPSS Statistics (Version 27; IBM Corp., Armonk, NY, USA) using the PROCESS macro for SPSS (Version 4.2; Hayes, 2022 [30]), and a two-sided *p* < 0.05 was considered statistically significant.

## 3. Results

### 3.1. Demographic Characteristics of Study Participants

The analytic sample comprised 1145 adults distributed across R1 (*n* = 138), R2 (*n* = 223), R3 (*n* = 417), and R4 (*n* = 367). Regional differences were evident in both FL and contextual characteristics (Table 1). Overall, FL was highest in R3 and lowest in R1, and the same general pattern was observed across the five FL domains. Mean age also varied modestly by region, with the highest average age in R4.

Socio-ecological scales also varied by region. Compared with R1, participants outside metropolitan areas generally reported higher importance, agreement, awareness, injunctive norms, and social norms, whereas dining preferences were highest in R2–R3 and lower in R4. Family support did not differ significantly by region.

These descriptive patterns suggest that smaller-city and county/rural settings were not uniformly disadvantaged; rather, they often combined stronger motivation- and norm-related supports with different structural conditions.

Regional differences were also observed in several demographic and socioeconomic indicators. Marriage was more common in R4, employment was highest in R1, higher education was more prevalent in R1–R2 than in R4, and income showed a clear gradient from metropolitan to county/rural settings (all *p* ≤ 0.001 except where noted in Table 1).

Health-related characteristics varied more modestly. Alcohol use increased from R1 to R4, smoking differed slightly by region, and chronic disease was less common in R3–R4 than in R1–R2. Physical activity also varied by region, although the pattern was not strictly monotonic.

Taken together, the descriptive results indicate meaningful regional variation in both FL and its socio-ecological context, supporting the rationale for the subsequent moderation analyses.

### 3.2. Measurement Properties of Socio-Ecological Constructs

Confirmatory factor analysis (CFA) supported the hypothesised eight-factor measurement model spanning intrapersonal, interpersonal, and environmental levels. Overall model fit was acceptable to good: χ^2^(436) = 1615.37, χ^2^/*df* = 3.71, GFI = 0.916, CFI = 0.947, TLI = 0.940, RMSEA = 0.049 (90% CI: 0.046–0.051), and SRMR = 0.050 (Table 2). Although the RMR was relatively elevated (0.098) and AGFI was marginally below the conventional 0.90 threshold (0.899), the remaining fit indices and residual-based diagnostics supported an adequately specified and well-fitting model.

Reliability and convergent validity were robust. Internal consistency was strong across all constructs (Cronbach’s α = 0.826–0.917), composite reliability met recommended criteria (CR ≥ 0.828), and convergent validity was supported (AVE = 0.546–0.706). During item screening, one awareness item with a negligible contribution was removed; the final scale retained satisfactory standardised loadings (0.671–0.803) and a coherent factor structure (Table 3).

Discriminant validity assessed via the heterotrait–monotrait ratio (HTMT) was generally adequate (<0.85) [25]. One conceptually adjacent pair—injunctive norms and social norms—showed a borderline HTMT of approximately 0.88; this is acceptable under the more liberal 0.90 criterion given their theoretical proximity (Table 4). Latent correlations across socio-ecological levels were moderate to high (intrapersonal–interpersonal r = 0.65; intrapersonal–environmental r = 0.66; interpersonal–environmental r = 0.79), consistent with related but non-redundant multi-level constructs.

### 3.3. Cross-Regional Comparability of the FL Measure

Additional multigroup CFA was conducted to assess cross-regional comparability of the FL instrument. Because the full multidimensional FL model showed unstable interfactor relations in some regional groups, invariance testing was performed separately for the five FL domains. Production, Selection, and Preparation showed clear support for configural, threshold, and metric invariance. Intake and Disposal each had just-identified configural baselines and were therefore interpreted more cautiously; Disposal showed no strong overall evidence of local noninvariance, whereas Intake showed some evidence of localized noninvariance under the constrained model. Overall, these findings support broad cross-regional comparability of the domain-level FL structure while indicating that the Intake and Disposal domains require more cautious interpretation. Detailed domain-specific measurement invariance results and score-test diagnostics are presented in Supplementary Tables S1 and S2.

### 3.4. Socio-Ecological Predictors of FL and Regional Moderation

In the fully adjusted model, several socio-ecological factors remained independently associated with overall FL after controlling for demographic, socioeconomic, and health-related covariates (Table 4). The strongest correlates were awareness, family support, and injunctive norms, while social norms and dining preferences also showed positive associations. Importance, agreement, family availability, and region as a main effect were not significant.

Robustness checks supported model interpretation. All VIF values were below 5, indicating no problematic multicollinearity, and the regional distribution of valid and invalid responses did not differ significantly. Detailed VIF values and sensitivity-analysis results are presented in Supplementary Tables S3 and S4, respectively.

Moderation was limited and predictor-specific. Significant Region × predictor interactions were observed for dining preferences, family support, and injunctive norms in the omnibus tests (Table 5). However, as shown below, the follow-up pattern was clearest for dining preferences and family support, whereas the injunctive-norm interaction was not supported by significant pairwise slope contrasts.

Interpretation: Omnibus interaction tests were significant for dining preferences, family support, and injunctive norms. Follow-up simple slopes and pairwise contrasts indicated clearer region-specific heterogeneity for dining preferences and family support than for injunctive norms.

### 3.5. Moderation by Region: Simple Slopes and Metropolises-Referenced Contrasts

Simple-slope analyses showed that dining preferences were positively associated with FL in all regions, with the strongest association in R4 and the weakest in R1 (Table 6). However, slope contrasts indicated limited between-region differentiation: only the rural–metropolis contrast (R4 > R1) was significant, while differences among R2–R4 were not.

Family support was also positively associated with FL across all regions and showed the clearest evidence of rural amplification. The R4 slope exceeded R1 and was also steeper than both R2 and R3, whereas R2 and R3 did not differ.

Injunctive norms showed consistently positive associations with FL across all regions. Although the omnibus interaction term was statistically significant, neither contrasts versus R1 nor contrasts among R2–R4 reached statistical significance. Accordingly, regional heterogeneity for injunctive norms was interpreted as limited in substantive terms (Table 7).

### 3.6. Regional Moderation of Socio-Ecological Determinants Across FL Components

Component-level analyses showed that regional moderation was concentrated in dining preferences for food intake and food choices/selection (Table 8). In both domains, dining preferences were positively associated with outcomes across all regions (Table 9). For food intake, the association was stronger in R2 and R4 than in R1, whereas differences within R2–R4 were not significant. For food choices/selection, only the R4–R1 contrast was significant (Table 10).

For family support, the clearest component-specific heterogeneity emerged for waste disposal. Positive associations were observed in all regions, but the effect was strongest in R4 (Table 9). The rural slope was steeper than those in R1, R2, and R3, whereas R2 and R3 did not differ (Table 10). Taken together, the component analyses suggest that preference-related heterogeneity was concentrated in food intake and food choices/selection, whereas family-support heterogeneity was most pronounced for waste disposal.

## 4. Discussion

This study showed that FL in Chinese adults was associated with multiple socio-ecological factors, with awareness, family support, and injunctive norms emerging as the strongest correlates after covariate adjustment. Region was not a significant main effect once compositional differences were controlled, indicating that average regional gaps in FL were less important than differences in how specific determinants operated across contexts.

Moderation was present but limited. Dining preferences and family support were positively associated with FL in all regions, yet the strength of these associations varied by settlement context. The clearest rural amplification was observed for family support, particularly in county/rural areas. Although the omnibus interaction for injunctive norms reached statistical significance, the absence of significant pairwise slope contrasts suggests that this pattern was not robust in substantive regional terms.

At the component level, heterogeneity was concentrated rather than diffuse. Dining preferences mattered most for food intake and food choices/selection, while family support was especially relevant for waste disposal. Because the Intake domain showed some evidence of localized noninvariance and the Disposal domain had a just-identified configural baseline, findings for these two domains should be interpreted somewhat more cautiously than those for the other FL domains.

These patterns are consistent with socio-ecological perspectives, which propose that food-related capability is shaped jointly by intrapersonal motivation, close social support, and wider normative environments [14,15,16,17]. The prominence of awareness suggests that practical understanding of healthy eating remains an important upstream resource, while the strong role of family support highlights that FL is not only an individual capability but also embedded in household routines and shared food practices.

The rural–urban differences are also practically meaningful. In county/rural areas, household support may matter more because food-related practices are more strongly organized around family routines, informal support, and shared domestic responsibilities. This interpretation is consistent with evidence from China showing that support-related processes can differ across rural and urban settings [18]. By contrast, the broadly similar slopes within R2–R4 for dining preferences suggest that preference-to-practice pathways may generalize across many non–tier-1 settings rather than following a simple linear urbanicity gradient.

These findings have implications for intervention design. In county/rural settings, FL promotion may benefit from household-oriented approaches that strengthen family support, shared meal planning, and practical waste-management routines. In non-tier-1 settings more broadly, helping people translate healthy food preferences into everyday food choices and intake may be especially valuable. At the same time, the consistently positive role of injunctive norms, coupled with limited robust regional differentiation, suggests that norm-aware communication strategies may be useful across regions.

This study has several strengths, including coverage across four regional tiers, a theory-driven socio-ecological framework, and multiple robustness checks. Additional domain-level measurement invariance analyses supported broad cross-regional comparability of the FL structure, VIF diagnostics indicated no problematic multicollinearity, and sensitivity analysis suggested that exclusion of invalid responses did not materially distort the regional composition of the analytic sample.

Several limitations should also be noted. First, the cross-sectional design precludes causal inference. Second, the use of snowball sampling through WeChat links and QR-code distribution may have favored adults with greater digital access, stronger online/social-network connectivity, and higher digital literacy [31]. The electronic survey format and the use of incentives may also have influenced participation differently across settlement contexts. Although the regional sensitivity analysis did not indicate a statistically significant distortion of the final regional composition, these recruitment features may still have affected participation patterns and limited representativeness. Third, the region was operationalized through a four-tier classification that necessarily simplifies within-tier heterogeneity in food environments.

Future work should extend these findings using probability-based or mixed-mode sampling, deeper measurement of local food environments, and longitudinal designs that can better assess how socio-ecological determinants of FL evolve across China’s diverse settlement contexts.

## 5. Conclusions

Socio-ecological determinants of FL were broadly robust across regions in China, but their magnitudes varied in modest and practically meaningful ways. The clearest context-specific pattern was a stronger family-support pathway in county/rural areas, particularly for waste-related practices. Region-sensitive strategies should, therefore, combine broad norm-aware communication with more household-oriented support in rural settings.

## Figures and Tables

**Table 1:** Participant Characteristics by Region. (**A**). Continuous variables (mean ± SD) and ANOVA. (**B**). Categorical variables (*n*, column %) and χ^2^ tests.

(**A**)							
Variable	R1 (*n* = 138)	R2 (*n* = 223)	R3 (*n* = 417)	R4 (*n* = 367)	Total (*N* = 1145)	F	*p*
Age (years)	36.38 ± 9.51	34.49 ± 11.20	36.64 ± 12.05	38.53 ± 12.45	36.80 ± 11.81	5.573	0.001
FL							
Production	83.53 ± 20.12	86.49 ± 20.56	88.84 ± 19.39	85.48 ± 21.77	86.66 ± 20.55	3.211	0.023
Selection	86.28 ± 20.24	90.21 ± 18.39	90.75 ± 18.13	87.92 ± 20.66	89.20 ± 19.32	2.636	0.049
Preparation/cooking	85.97 ± 20.53	92.43 ± 19.70	94.01 ± 15.55	90.28 ± 19.70	91.54 ± 18.56	7.232	<0.001
Intake	82.84 ± 26.35	90.75 ± 22.16	94.27 ± 17.57	89.25 ± 21.32	90.60 ± 21.19	9.847	<0.001
Disposal	87.68 ± 22.56	89.10 ± 20.33	92.62 ± 17.67	88.71 ± 20.98	90.09 ± 19.98	3.947	0.008
FL—Overall index	85.02 ± 18.43	89.88 ± 17.32	92.18 ± 15.57	88.36 ± 19.28	89.64 ± 17.65	6.967	<0.001
Socio-ecological scales							
Dining preferences	3.63 ± 0.87	3.85 ± 0.77	3.80 ± 0.65	3.60 ± 0.82	3.73 ± 0.76	7.373	<0.001
Importance	3.38 ± 0.95	3.49 ± 0.87	4.24 ± 1.11	4.28 ± 1.27	4.00 ± 1.17	53.982	<0.001
Agreement	3.59 ± 1.31	4.09 ± 1.23	3.97 ± 0.97	3.73 ± 0.94	3.87 ± 1.07	8.319	<0.001
Awareness	6.62 ± 1.84	7.24 ± 1.56	7.82 ± 1.65	7.68 ± 1.88	7.52 ± 1.78	19.032	<0.001
Family support	3.59 ± 0.99	3.58 ± 0.95	3.62 ± 0.89	3.54 ± 0.91	3.58 ± 0.92	0.568	0.636
Injunctive norms	3.47 ± 0.98	3.82 ± 0.90	4.01 ± 0.66	3.87 ± 0.79	3.86 ± 0.81	13.829	<0.001
Social norms	3.46 ± 0.95	3.82 ± 0.92	4.00 ± 0.62	3.87 ± 0.76	3.86 ± 0.79	14.33	<0.001
Family availability	3.74 ± 1.03	3.81 ± 0.91	3.93 ± 0.81	3.75 ± 0.92	3.83 ± 0.90	3.443	0.017
(**B**)							
Variable	R1 (*n* = 138)	R2 (*n* = 223)	R3 (*n* = 417)	R4 (*n* = 367)	χ^2^ (*df*)	*p*	Cramér’s V
Marital status, Married	86 (62.3%)	120 (53.8%)	248 (59.5%)	255 (69.5%)	16.19 (3)	0.001	0.119
Employment, Employed	123 (89.1%)	161 (72.2%)	295 (70.7%)	199 (54.2%)	63.52 (3)	<0.001	0.236
Education, ≤High school	48 (34.8%)	60 (26.9%)	168 (40.3%)	206 (56.1%)	54.35 (3)	<0.001	0.218
Alcohol use							
No	51 (37.0%)	90 (40.4%)	201 (48.2%)	207 (56.4%)	22.35 (3)	<0.001	0.14
≥Once	87 (63.0%)	133 (59.6%)	216 (51.8%)	160 (43.6%)			
Smoking							
No	80 (58.0%)	156 (70.0%)	305 (73.1%)	251 (68.4%)	11.37 (3)	0.01	0.1
≥Once	58 (42.0%)	67 (30.0%)	112 (26.9%)	116 (31.6%)			
Chronic disease, Yes	51 (37.0%)	66 (29.6%)	49 (11.8%)	55 (15.0%)	62.44 (3)	<0.001	0.234
Physical activity							
Inactive	10 (7.2%)	12 (5.4%)	16 (3.8%)	21 (5.7%)			
Low activity	23 (16.7%)	51 (22.9%)	65 (15.6%)	62 (16.9%)	64.22 (12)	<0.001	0.137
Moderate activity	29 (21.0%)	56 (25.1%)	155 (37.2%)	164 (44.7%)			
Active	42 (30.4%)	81 (36.3%)	139 (33.3%)	84 (22.9%)			
Very active	34 (24.6%)	23 (10.3%)	42 (10.1%)	36 (9.8%)			
Monthly income (Yuan)							
≤5000	21 (15.2%)	72 (32.3%)	191 (45.9%)	255 (69.5%)	171.78 (6)	<0.001	0.274
5001–10,000	45 (32.6%)	59 (26.5%)	125 (30.0%)	66 (18.0%)			
≥10,001	72 (52.2%)	92 (41.3%)	101 (24.2%)	46 (12.5%)			

Notes. Values for categorical variables are *n* (column %); percentages are calculated within region (denominator = regional *n*). Two-sided tests with α = 0.05. For categorical comparisons, statistics are Pearson’s χ^2^ with degrees of freedom in parentheses and effect size Cramér’s V.

**Table 2:** Overall model fit indices for the confirmatory factor analysis (CFA).

Index	Criterion	Observed
χ^2^ (*df*)	—	1615.37 (436)
χ^2^/*df*	<5	3.705
GFI/AGFI	>0.90	0.916/0.899
CFI/TLI (NNFI)	>0.90 (prefer >0.95)	0.947/0.940
NFI/IFI	>0.90	0.930/0.948
RMSEA (90% CI)	<0.06–0.08	0.049 (0.046–0.051)
SRMR/RMR	<0.08/<0.05	0.050/0.098
PGFI/PNFI/PCFI	>0.50	0.757/0.817/0.833

**Table 3:** Socio-ecological measurement properties (CFA-refined).

Factors	Example	Item	CITC	Factorload	Cross-Load	Cronbach’s α	AVE	CR
Intrapersonal Level								
Dining preferences	The restaurant’s dishes satisfy all five senses.	4	0.626	0.69	>0.20	0.826	0.546	0.828
	The dishes are made from natural ingredients.		0.684	0.777				
	Dishes are better for health.		0.686	0.759				
	Dishes can help regulate weight.		0.613	0.708				
Motivation importance	How important is a healthy diet to you for reducing the risk of diseases or disorders?	5	0.754	0.763	>0.20	0.917	0.692	0.918
	How important is a healthy diet to you for improving your mood through your body?		0.821	0.864				
	How important is a healthy diet to you for weight control?		0.819	0.866				
	How important is a healthy diet to you for increasing concentration?		0.745	0.765				
	How important is a healthy diet to you for feeling energetic?		0.799	0.829				
Motivation, agreement	Do you agree that a healthy diet can reduce the risk of certain diseases or disorders	5	0.742	0.825	>0.20	0.899	0.648	0.901
	Do you agree that a healthy diet can improve your mood through your body?		0.826	0.896				
	Do you agree that a healthy diet can help with weight control?		0.822	0.900				
	Do you agree that a healthy diet (e.g., not skipping any meals) can increase concentration?		0.742	0.589				
	Do you agree that a healthy diet can make each day full of energy?		0.619	0.803				
Motivation, awareness	How important is a healthy diet life to you?	6	0.650	0.671	>0.20	0.824	0.564	0.866
	How confident are you in maintaining a healthy diet life?		0.668	0.763				
	How prepared are you for maintaining a healthy diet life?		0.651	0.741				
	How important is it to you to cook food healthily?		0.683	0.719				
	How confident are you in cooking food healthily?		0.688	0.803				
	How prepared are you for cooking food healthily?		0.272		<0.20	Delete		
Interpersonal Level								
Injunctive norm	Family members think I should engage in a healthy diet life.	4	0.667	0.752	>0.20	0.855	0.597	0.856
	Friends think I should engage in a healthy diet life		0.704	0.774				
	Experts (doctors, nutritionists, etc.) think I should engage in a healthy diet life.		0.705	0.773				
	Classmates or colleagues believe I should engage in a healthy diet life.		0.717	0.789				
Family support	Healthy nutritious dinners are prepared by me or my family	3	0.700	0.772	>0.20	0.863	0.684	0.866
	Family members help me to consume fruits and vegetables		0.759	0.841				
	Family members prepare healthy snacks or meals together		0.764	0.861				
Environmental Level								
Social norms	The government thinks I should engage in a healthy diet life	4	0.669	0.708	>0.20	0.848	0.585	0.849
	TV programs (including those watched online) think I should engage in a healthy diet life		0.704	0.767				
	Newspapers and magazines think I should engage in a healthy diet life		0.715	0.739				
	Online information (Weibo, TikTok, etc.) thinks I should engage in a healthy diet life		0.661	0.691				
Family availability	Fresh fruits or vegetables are available at home	2	0.706	0.846	>0.20	0.828	0.706	0.828
	Healthy snacks are available at home.		0.706	0.854				

Notes: standardized loadings shown as ranges. AVE = average variance extracted; CR = composite reliability.

**Table 4:** General Linear Model Predicting FL: Main Effects with Covariates.

Source	*df*	F	*p*	Partial η^2^
Covariates				
Age	1	0.045	0.813	0.000
Marital status	1	2.604	0.107	0.002
Occupation	1	1.126	0.289	0.001
Education level	1	7.777	0.005	0.007
Gender	1	1.593	0.207	0.001
Alcohol use	1	6.775	0.009	0.006
Smoking	1	0.466	0.495	0.001
Chronic disease	1	4.071	0.044	0.004
Income	1	1.776	0.183	0.002
Physical activity	1	1.887	0.170	0.002
BMI	1	0.934	0.334	0.001
Intrapersonal level				
Dining preferences (Z)	1	38.605	<0.001	0.034
Importance (Z)	1	0.247	0.619	0.000
Agreement (Z)	1	0.002	0.961	0.000
Awareness (Z)	1	63.868	<0.001	0.055
Interpersonal level				
Family support (Z)	1	77.627	<0.001	0.066
Injunctive norms (Z)	1	42.276	<0.001	0.037
Environmental level				
Social norms (Z)	1	14.713	<0.001	0.013
Family availability (Z)	1	2.375	0.124	0.002
Region (4 levels)	3	2.005	0.112	0.005

Model fit: *N* = 1145; R^2^ = 0.629; adjusted R^2^ = 0.614; error *df* = 1099; MSE = 119.703. Notes: Dependent variable = FL. Predictors labeled “(Z)” are standardized (mean-centered, SD = 1). Partial η^2^ reported.

**Table 5:** Regional Moderation (Region × Predictor) on FL.

Interaction Term	*df*	F	*p*	Partial η^2^
Region × Dining preferences (Z)	3	4.824	0.002	0.013
Region × Importance (Z)	3	1.514	0.209	0.004
Region × Agreement (Z)	3	1.219	0.301	0.003
Region × Awareness (Z)	3	2.165	0.091	0.006
Region × Family support (Z)	3	3.813	0.010	0.01
Region × Injunctive norms (Z)	3	4.148	0.006	0.011
Region × Social norms (Z)	3	1.794	0.147	0.005
Region × Family availability (Z)	3	1.182	0.315	0.003

Model fit: *N* = 1145; same controls as Table 4; Type III SS; partial η^2^ reported.

**Table 6:** Simple slopes (unstandardized b) of socio-ecological predictors on FL by Region (PROCESS, HC3).

Predictor	Region	b (SE)	t	*p*	95% CI
Dining preferences (Z)	R1 (ref.)	5.535 (1.288)	4.30	<0.001 ***	[3.008, 8.063]
	R2	7.898 (1.442)	5.48	<0.001 ***	[5.069, 10.727]
	R3	7.388 (1.457)	5.07	<0.001 ***	[4.530, 10.246]
	R4	9.567 (1.226)	7.81	<0.001 ***	[7.162, 11.972]
Family support (Z)	R1 (ref.)	6.405 (1.509)	4.24	<0.001 ***	[3.444, 9.366]
	R2	8.019 (1.405)	5.71	<0.001 ***	[5.263, 10.775]
	R3	8.723 (0.948)	9.21	<0.001 ***	[6.864, 10.582]
	R4	11.835 (1.113)	10.64	<0.001 ***	[9.652, 14.018]
Injunctive norms (Z)	R1 (ref.)	11.824 (1.062)	11.14	<0.001 ***	[9.741, 13.907]
	R2	10.421 (0.870)	11.98	<0.001 ***	[8.714, 12.128]
	R3	9.805 (0.944)	10.38	<0.001 ***	[7.952, 11.658]
	R4	11.431 (1.077)	10.61	<0.001 ***	[9.317, 13.544]

Notes. Outcome = FL. Entries are unstandardized coefficients with HC3 SEs from PROCESS v4.2 (Model 1). Models adjust for prespecified covariates (age, marital status, occupation, education, gender, alcohol, smoking, chronic disease, income, physical activity, BMI, and other socio-ecological factors). Significance: *** *p* < 0.001.

**Table 7:** Pairwise slope contrasts by region (PROCESS Model 1, HC3).

Predictor	Contrast	Δb (SE)	Δt	Δ*p*
Dining preferences (Z)	R2 − R1	2.362 (1.945)	1.21	0.225
	R3 − R1	1.853 (1.937)	0.96	0.339
	R4 − R1	4.032 (1.795)	2.25	0.025 *
	R3 − R2	−0.036 (1.1227)	−0.03	0.974
	R4 − R2	1.165 (1.1065)	1.05	0.293
	R4 − R3	1.201 (1.1452)	1.05	0.294
Family support (Z)	R2 − R1	1.614 (2.077)	0.78	0.437
	R3 − R1	2.318 (1.769)	1.31	0.19
	R4 − R1	5.430 (1.856)	2.93	0.004 **
	R3 − R2	0.704 (1.6812)	0.42	0.676
	R4 − R2	3.816 (1.7866)	2.14	0.033 *
	R4 − R3	3.112 (1.4364)	2.17	0.031 *
Injunctive norms (Z)	R2 − R1	−1.403 (1.349)	−1.04	0.299
	R3 − R1	−2.019 (1.435)	−1.41	0.16
	R4 − R1	−0.393 (1.510)	−0.26	0.795
	R3 − R2	−0.616 (1.2863)	−0.48	0.632
	R4 − R2	1.009 (1.3701)	0.74	0.461
	R4 − R3	1.626 (1.4141)	1.15	0.25

Notes. Δb is the slope difference between regions; positive values indicate a steeper slope in the first-listed region. R1 corresponds to Metropolises (Tier-1). Robust SEs are HC3. Significance: *p* < 0.05 (*), *p* < 0.01 (**).

**Table 8:** Region moderation (X × Region) for five components of FL.

Predictor	Outcome (FL Component)	ΔR^2^ (X × Region)	F	*p*
Dining preferences	Food production	0.004	1.494	0.214
	Food choices	0.006	3.191	0.023
	Preparation & cooking	0.002	1.308	0.270
	Food intake	0.008	3.559	0.014
	Waste disposal	0.003	2.205	0.086
Family support	Food production	0.003	1.232	0.297
	Food choices	0.006	2.500	0.058
	Preparation & cooking	0.001	0.419	0.740
	Food intake	0.001	0.567	0.637
	Waste disposal	0.008	3.886	0.009

Notes: ΔR^2^ is the change in R^2^ attributable to the interaction; F uses heteroscedasticity-consistent HC3. The region has four levels: Metropolises (Tier-1), Tier-2, Small/Medium cities, Rural.

**Table 9:** Simple slopes of X predicting FL components at each Region level.

Predictor	Outcome	Region	b	SE	t	*p*	95% CI
Dining preferences	Food intake	R1 (ref.)	4.879	1.847	2.64	0.008	[1.256, 8.502]
		R2	10.194	1.634	6.24	<0.001	[6.988, 13.400]
		R3	6.753	1.547	4.37	<0.001	[3.718, 9.788]
		R4	10.129	1.317	7.69	<0.001	[7.545, 12.712]
	Food choices	R1 (ref.)	4.332	1.615	2.68	0.007	[1.164, 7.501]
		R2	7.474	1.750	4.27	<0.001	[4.041, 10.908]
		R3	6.744	1.531	4.40	<0.001	[3.740, 9.749]
		R4	9.890	1.302	7.60	<0.001	[7.336, 12.443]
Family support	Waste disposal	R1 (ref.)	4.396	1.908	2.30	0.021	[0.653, 8.139]
		R2	4.684	1.593	2.94	0.003	[1.560, 7.809]
		R3	7.221	1.086	6.65	<0.001	[5.090, 9.353]
		R4	11.122	1.195	9.30	<0.001	[8.777, 13.468]

**Table 10:** Regional slope contrasts for FL components (PROCESS Model 1, HC3).

Predictor	Outcome	Contrast	Δb (SE)	Δt	Δ*p*
Dining preferences	Food intake	R2 − R1	5.315 (2.457)	2.16	0.031 *
		R3 − R1	1.874 (2.407)	0.78	0.436
		R4 − R1	5.250 (2.285)	2.3	0.022 *
		R3 − R2	−3.441 (2.2274)	−1.55	0.123
		R4 − R2	−0.065 (2.0796)	−0.03	0.975
		R4 − R3	3.375 (2.0094)	1.68	0.093
Dining preferences	Food choices	R2 − R1	3.142 (2.383)	1.32	0.188
		R3 − R1	2.412 (2.209)	1.09	0.275
		R4 − R1	5.558 (2.087)	2.66	0.008 **
		R3 − R2	−0.729 (2.3057)	−0.32	0.752
		R4 − R2	2.415 (2.1758)	1.11	0.267
		R4 − R3	3.145 (1.9909)	1.58	0.115
Family support	Waste disposal	R2 − R1	0.288 (2.483)	0.12	0.908
		R3 − R1	2.825 (2.170)	1.3	0.193
		R4 − R1	6.726 (2.229)	3.02	0.003 **
		R3 − R2	2.537 (1.8942)	1.34	0.181
		R4 − R2	6.437 (1.9724)	3.26	0.001 **
		R4 − R3	3.900 (1.5844)	2.46	0.014 *

Notes. Δb is the slope difference between regions; positive values indicate a steeper X → Outcome association in the first-listed region. R1 corresponds to Metropolises (Tier-1). Robust SEs are HC3. Significance: *p* < 0.05 (*), *p* < 0.01 (**).

## Data Availability

The data are not publicly available due to privacy; access to the data can be requested from the corresponding author.

## Supplementary Materials

The following supporting information can be downloaded at https://www.mdpi.com/article/10.3390/nu18071151/s1. Supplementary Table S1: Domain-specific measurement invariance of the food literacy scale across four regional groups (R1–R4). Supplementary Table S2: Score-test diagnostics for FL domains with just-identified configural models. Supplementary Table S3: Variance inflation factors for predictors in the fully adjusted FL model. Supplementary Table S4: Sensitivity analysis of regional distribution between valid and invalid responses.
