# Supplementary material for: Socio-Ecological Correlates of Food Literacy Across Regional Contexts in China
Source: Nutrients. 2026 Apr 3;18(7):1151. doi: 10.3390/nu18071151 (PMC13074418; doi:10.3390/nu18071151)
Supplement: Supplementary file 1 [file nutrients-18-01151-s001.zip › nutrients-4219437-supplementary.pdf]

Supplementary Materials

Regional Variation in Socio-Ecological Correlates of Food Literacy in China

Suggested in-manuscript back-matter statement:

Supplementary Materials: The following supporting information can be downloaded at: [to be inserted by the journal during production], Table S1: Domain-specific measurement invariance of the food literacy scale across four regional groups (R1–R4); Table S2: Score-test diagnostics for FL domains with just-identified configural models; Table S3: Variance inflation factors for predictors in the fully adjusted FL model; Table S4: Sensitivity analysis of regional distribution between valid and invalid responses.

Formatting note. Table captions are placed above each table, and explanatory notes are placed below each table, consistent with MDPI table formatting practice.

Table S1. Domain-specific measurement invariance of the food literacy scale across four regional groups (R1–R4).

Domain	Model	χ^2	df	CFI	RMSEA	SRMR	ΔCFI	ΔRMSEA	Interpretation
Production	Configural	208.256	56	0.997	0.098	0.039	—	—	Baseline model acceptable
Production	Threshold	330.047	98	0.996	0.091	0.039	-0.001	-0.007	Threshold invariance supported
Production	Metric	349.452	116	0.996	0.084	0.040	0.000	-0.007	Metric invariance supported
Selection	Configural	72.523	20	0.997	0.096	0.037	—	—	Baseline model acceptable
Selection	Threshold	170.631	50	0.993	0.092	0.037	-0.004	-0.004	Threshold invariance supported
Selection	Metric	204.607	62	0.992	0.090	0.043	-0.001	-0.002	Metric invariance supported
Preparation	Configural	119.779	56	0.998	0.063	0.038	—	—	Baseline model acceptable
Preparation	Threshold	222.520	98	0.996	0.067	0.038	-0.002	0.004	Threshold invariance supported
Preparation	Metric	265.744	116	0.996	0.067	0.040	0.000	0.000	Metric invariance supported
Intake	Configural	0.000	0	1.000	0.000	0.000	—	—	Just-identified baseline
Intake	Threshold	65.999	18	0.994	0.097	0.000	-0.006	0.097	Constrained model acceptable; interpret cautiously
Intake	Metric	77.907	24	0.994	0.089	0.016	0.000	-0.008	Metric model acceptable; interpret cautiously
Disposal	Configural	0.000	0	1.000	0.000	0.000	—	—	Just-identified baseline
Disposal	Threshold	37.731	18	0.998	0.062	0.000	-0.002	0.062	Constrained model acceptable; interpret cautiously
Disposal	Metric	48.962	24	0.998	0.060	0.017	0.000	-0.002	Metric model acceptable; interpret cautiously

Note. *Measurement invariance was examined separately for the five food literacy (FL) domains using multigroup confirmatory factor analysis with ordered categorical indicators. Configural, threshold, and metric invariance were tested sequentially. Invariance decisions were based primarily on changes in CFI and RMSEA between nested models. The Intake and Disposal domains each consisted of three indicators; therefore, their configural models were just-identified (df = 0), and comparisons involving the configural baseline should be interpreted cautiously.*

Table S2. Score-test diagnostics for FL domains with just-identified configural models.

Domain	Constrained model	Total score test χ^2	df	p-value	Interpretation
Intake	Metric	77.604	45	0.002	Some evidence of localized noninvariance
Disposal	Metric	48.781	45	0.324	No strong overall evidence of local noninvariance

Note. *Score-test diagnostics were examined for the Intake and Disposal domains because their configural models were just-identified (df = 0), which limited the informativeness of baseline fit comparisons. For Disposal, the overall score test was not significant, suggesting no strong evidence of local noninvariance under the constrained model. For Intake, the overall score test was significant, indicating some localized noninvariance; accordingly, findings for this domain should be interpreted with greater caution.*

Table S3. Variance inflation factors for predictors in the fully adjusted FL model.

Predictor	VIF	Interpretation
Dining preferences	1.394	Acceptable
Importance	1.463	Acceptable
Agreement	1.499	Acceptable
Awareness	1.753	Acceptable
Family support	1.951	Acceptable
Injunctive norms	3.568	Acceptable
Social norms	3.485	Acceptable
Family availability	1.992	Acceptable
Age	1.573	Acceptable
Marital status	1.316	Acceptable
Occupation	1.355	Acceptable
Education	1.444	Acceptable
Gender	1.441	Acceptable
Alcohol use	1.469	Acceptable
Smoking	1.626	Acceptable
Disease	1.214	Acceptable
Income	1.337	Acceptable
Physical activity	1.107	Acceptable
R2	2.217	Acceptable
R3	3.008	Acceptable
R4	3.204	Acceptable

Note. *Variance inflation factors (VIFs) were examined to assess potential multicollinearity in the fully adjusted FL model. Region was dummy-coded with R1 as the reference group. All VIF values were below the conventional threshold of 5, indicating no problematic multicollinearity.*

Table S4. Sensitivity analysis of regional distribution between valid and invalid responses.

Region	Invalid, n (%)	Valid, n (%)	Total, n (%)
R1	14 (9.2)	138 (90.8)	152 (100.0)
R2	19 (7.9)	223 (92.1)	242 (100.0)
R3	26 (5.9)	417 (94.1)	443 (100.0)
R4	45 (10.9)	367 (89.1)	412 (100.0)
Total	104 (8.3)	1145 (91.7)	1249 (100.0)

Note. *Regional distribution did not differ significantly between valid and invalid responses (Pearson $\chi^2 = 7.369$, $df = 3$, $p = 0.061$). Three cases with missing region information were not included in this comparison.*
